# A fuzzy reliability assessment methodology for city gas stations based on an extended T-S fault tree

**DOI:** 10.1016/j.heliyon.2024.e34641

**Published:** 2024-07-14

**Authors:** Daqing Wang, Ping Liang, Tingting Luo, Haihong Yu

**Affiliations:** a School of Petroleum Engineering, Chongqing University of Science & Technology, Chongqing, 401331, China; b Chongqing Key Laboratory of Complicated Oil & Gas Exploration and Development, Chongqing University of Science & Technology, Chongqing, 401331, China

**Keywords:** Extended T-S fault tree, Bayesian updating estimation, Fuzzy group decision-making, Reliability assessment, City gas station

## Abstract

City gas stations (CGSs) play a crucial role in ensuring a stable and safe supply of natural gas to urban users. However, as the service time of stations increases and the performance of components deteriorates, concerns about the safety and reliability of these station have grown among operators and local government authorities. This paper proposes a fuzzy reliability assessment methodology for CGSs that considers the polymorphism of component faults and the uncertainties associated with fault relationships, failure probabilities, and fault magnitudes. The methodology utilizes T-S fuzzy gates to describe the correlation among events and constructs a T-S fuzzy fault tree for CGSs. Component fault states are represented using fuzzy numbers, and a fuzzy group decision-making approach is introduced to evaluate the current fault magnitude of components. To handle the uncertainty caused by sparse failure sample data, a Bayesian updating estimation method is presented to estimate the failure probabilities of components. Furthermore, T-S fuzzy importance analysis is applied to identify the weak points in the CGS system. The effectiveness of the developed methodology is demonstrated through a case study of reliability analysis of a city gas distribution station. The research findings provide valuable support for optimizing the design and implementing preventive maintenance of CGSs.

## Highlights

• A workflow of extended T-S fault tree is developed for CGS reliability assessment.
• Bayesian updating method estimates failure probabilities with limited sample data.
• T-S fuzzy importance analysis identifies weak points in the CGS system.
• The methodology facilitates optimize design and implement preventive maintenance.

## 1 Introduction

Currently, the penetration rate of urban piped gas in China has exceeded 80 %. The gas distribution pipeline network is extensive and is transitioning from large-scale construction to comprehensive operation and maintenance. City gas stations (CGSs) are crucial hubs in the gas distribution network, playing a vital role in providing stable and safe gas supply to urban users [1,2]. However, as the stations age and their performance deteriorate, the challenges of operational safety and maintenance will increase. The failure of one or more components in the CGS system could jeopardize the safety of the entire station, disrupt gas supply, or even lead to a major hazardous accident. For instance, in 2004, a natural gas factory explosion in Belgium resulted in the death of fourteen individuals and left over two hundred injured. Similarly, in 2009, a gas leakage-induced explosion caused the largest fire in Moscow since World War II [3]. Therefore, regular reliability analysis and evaluation of the CGS system are necessary to identify and eliminate potential hazards and ensure safe operation.

There are various methods available for assessing safety and risk in the process industries. For example, fault tree analysis (FTA) [[4], [5], [6]] and Bow-tie analysis (BTA) [7,8], which are based on Boolean algebra and probability theory, are widely utilized. These methods are crucial role in identifying potential risk sources and ensuring the safety and reliability of complex process systems. However, the classical FTA and BTA techniques have limitations such as their static structure, lack of consideration for event independency, and reliance on precise failure data, which restrict their widespread and deeper application. To address the uncertainty arising from the lack, scarcity, or inaccuracy of data, Tanaka et al. [9] introduced fuzzy set theory into FTA. They replaced the failure probability of each component with fuzzy possibility and calculated the fuzzy possibility of the occurrence of the top event through fuzzy operations. Lavasani et al. [10] expanded the fuzzy fault tree analysis (FFTA) methodology to the petrochemical process industry, where fire, explosions, and toxic gas releases are acknowledged as potential hazards. Dong and Yu [11], Wang et al. [12], Cheliyan and Bhattacharyya [13] integrated expert elicitation with FFTA to evaluate the fuzzy probabilities of accidents in oil and gas production systems and to identify system vulnerabilities. Yazdi et al. [14] conducted a comprehensive review of uncertainty handling methods in FTA-based risk assessment, with a particular emphasis on how assessors can handle uncertainty based on the available evidence and use it as an input to FTA. Ferdous et al. [15] introduced fuzzy-based and evidence theory-based BTA approaches, along with a sensitivity analysis technique, to address data uncertainty, interdependence of input events, and mitigate risks. Das et al. [16] proposed a novel approach called fuzzy-2 BTA, which integrates bow-tie (BT) with type II fuzzy set (T2FS) to accurately quantify the risk and consequences of an undesired event. This method effectively addresses the uncertainty in experts' opinions and membership functions when assessing the probabilistic assessment of the basic events (BEs) by utilizing T2FS.

However, the FFTA methods mentioned in references 9 to 16 have two significant limitations: (1) they do not account for the polymorphism of component faults and their effects on the system, and (2) the logic gates are rigid and do not allow for the inclusion of linguistic information, such as operational experiences or experts’ knowledge. To address these limitations, Song et al. [17] developed the T-S (Takagi & Sugeno) model-based FFTA method. This approach utilizes fuzzy numbers to describe fault probability and magnitude and constructs fuzzy gates using the T-S model to depict the relationships among events. Building upon the work of Song et al. Yao and Zhao [18] applied the T-S FFTA method to conduct reliability research on hydraulic systems. Additionally, Yao et al. [19] extended the traditional component importance analysis methods of FTA to T-S FFTA, introducing the concept of T-S importance and its calculation method. In a subsequent study, Yao et al. [20] proposed a reliability optimization method based on T-S fault tree (FT) and EPSO algorithm. This method constructed a reliability optimization model for multi-state systems, considering constraint conditions such as system cost, weight, and volume. Sun [21], Lei [22], Li [23], and Zhou [24] et al. conducted reliability analysis and fault diagnosis on systems and devices from different domains, such as avionics full duplex switched ethernet (AFDX), automatic transmission, and tunnel detection robots, based on the T-S FFTA method.

To mitigate subjective errors resulting from excessive dependence on expert knowledge, Wu et al. [25] proposed an enhanced T-S FFTA method that incorporates the importance indexes of BEs obtained through Monte Carlo (MC) simulation as a fundamental basis for constructing the improved T-S gate rules. Additionally, Bi et al. [26], Chen et al. [27], and Zhang et al. [28] have applied T-S FT to construct Bayesian networks (BN) for the analysis of system reliability. This approach has helped to overcome some of the shortcomings of classical FT, such as the inability to handle the ambiguity of fault linkages among events and the polymorphism of faults when converting them into BN.

Given the distinctive characteristics of process systems, including their high level of systematicity, clear system hierarchy, and ambiguity in fault linkages and fault severity, the utilization of T-S FFT has proven to be an effective method for analyzing the safety and reliability of these systems. However, it is crucial to acknowledge other objective realities for CGSs, such as the scarcity of on-site failure data and the challenges associated with accurately measuring component fault states. To address these challenges, this study aims to develop an extended T-S FFTA methodology that incorporates the T-S FT with Bayesian updating estimation and fuzzy group decision-making to enhance the assessment of CGS reliability. Section 2 provides a comprehensive explanation of the complete methodology. In Section 3, a case study is presented to demonstrate the applicability of the proposed methodology. Finally, Section 4 summarizes the conclusions drawn from the research.

## 2 Preliminaries

This section will review some basic concepts related to the proposed fuzzy reliability assessment methodology for CGSs. These concepts include the T-S fault tree, fuzzy group decision making, and Bayesian estimation.

### 2.1 T-S fault tree

**Fig. 1:**
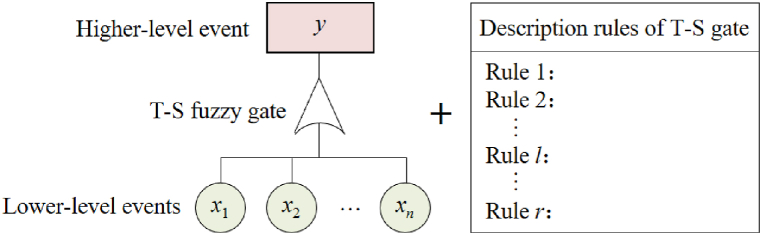
T-S fuzzy gate and its description rules.

A T-S fault tree consists of T-S fuzzy gates and multi-level events, as shown in Fig. 1 [20]. The T-S fuzzy gate is constructed based on the T-S fuzzy model proposed by Takagi and Sugeno [29], which incorporates a series of IF-THEN fuzzy rules. By using T-S fuzzy gates instead of traditional logic gates in the FT, the relationships between lower-level events and higher-level events can be depicted, effectively addressing the uncertainties associated with failure mechanisms and event linkages.

The description rules for T-S fuzzy gate are presented in Table 1 [30]. Given fuzzy rules *l* (*l* = 1, 2, …, *r*), if the fault states of lower-level events $x_{i}\left(i=1,2,\cdots ,n\right)$ are $S_{i}^{\left(a_{i}\right)}\;\left(a_{i}=1,2,\cdots ,k_{i}\right)$, the occurrence probabilities of higher-level event *y* with fault states $S_{y}^{\left(b_{y}\right)}\;\left(b_{y}=1,2,\cdots ,k_{y}\right)$ are $P_{\left(l\right)}\left(y=S_{y}^{\left(b_{y}\right)}\right)$. Here, *n* represents the quantity of lower-level events; *k*_*y*_ denotes the number of fault states of higher-level event *y*; *r* stands for the total number of rules, $r=\prod_{i=1}^{n}k_{i}=k_{1}k_{2}\cdots k_{n}$; $0\leq S_{i}^{\left(a_{i}\right)}\leq 1\;\left(a_{i}=1,2,\cdots ,k_{i}\right)$ represents various operational states (such as normal, mediated, and failed) of lower-level events $x_{i}\left(i=1,2,\cdots ,n\right)$; $0\leq S_{y}^{\left(b_{y}\right)}\leq 1\;\left(b_{y}=1,2,\cdots ,k_{y}\right)$ represents the different states (e.g., normal, mediated, and failed) of the higher-level event *y*.

**Table 1:** Description rules of T-S fuzzy gate.

Rules	*x*_1_	*x*_2_	···	*x*_n_	*y*			
					$S_{y}^{\left(1\right)}$	$S_{y}^{\left(2\right)}$	···	$S_{y}^{\left(k_{y}\right)}$
*l*	$S_{1}^{\left(a_{1}\right)}$	$S_{2}^{\left(a_{2}\right)}$	···	$S_{n}^{\left(a_{n}\right)}$	$P_{\left(l\right)}\left(y=S_{y}^{\left(1\right)}\right)$	$P_{\left(l\right)}\left(y=S_{y}^{\left(2\right)}\right)$	···	$P_{\left(l\right)}\left(y=S_{y}^{\left(k_{y}\right)}\right)$

The T-S fault tree allows for quantitative analysis when reliability data for BEs is available, enabling calculations of the probability of top event occurrence and the importance of BEs as reliability indicators.

### 2.2 Fuzzy group decision making

Group decision-making involves the consolidation and processing of preference information provided by individual decision-makers, which includes subjective probabilities, rankings, binary comparisons, preference utilities, and other relevant factors. However, evaluating this information poses challenges due to uncertainty and fuzziness. To address this fuzziness, the application of fuzzy set theory offers an effective approach in the decision-making process [31].

**Fig. 2:**
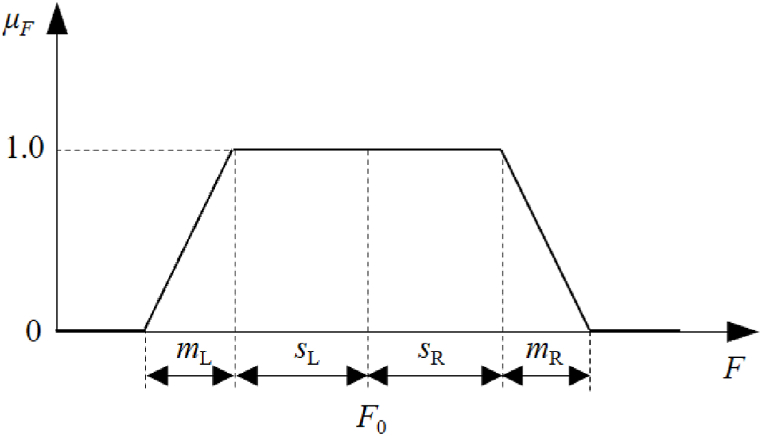
Fuzzy number represented by trapezoidal membership function.

In the context of group decision-making, decision-makers' opinions and preferences can be expressed as fuzzy numbers, where the degree of membership indicates the strength of their beliefs or preferences. To facilitate the representation of fuzzy numbers, a commonly used trapezoidal membership function is employed. This function is illustrated in Fig. 2 and defined by Equation (1), with the abbreviation “$F\equiv \left(F_{0},s_{L},m_{L},s_{R},m_{R}\right)$” [17]. When $s_{L}=s_{R}=0$, the trapezoidal membership function simplifies to a triangular membership function. The condition $m_{L}=m_{R}=0$ signifies a closed interval. Furthermore, when $s_{L}=s_{R}=0$ and $m_{L}=m_{R}=0$, the fuzzy number transitions into a crisp value.

(1) $$\mu_{F}=\left\{ \begin{array}{c} \frac{F-\left(F_{0}-s_{L}-m_{L}\right)}{m_{L}},F_{0}-s_{L}-m_{L}<F\leq F_{0}-s_{L}\\ 1,F_{0}-s_{L}<F\leq F_{0}+s_{R}\\ \frac{F_{0}+s_{R}+m_{R}-F}{m_{R}},F_{0}+s_{R}<F\leq F_{0}+s_{R}+m_{R}\\ 0,others \end{array}\right.$$

Due to variations in expertise levels and field experience among experts, conflicts and consensus may arise during the group decision-making process. Therefore, it is crucial to identify a rational approach to consolidate the ambiguous opinions of individuals into a collective consensus. Several aggregation methods have been proposed thus far, such as the similarity aggregation method (SAM) [32], the consistency aggregation method (CAM) [12,31], and the fuzzy TOPSIS method [33]. The selection of the most appropriate method should consider the specific characteristics of the panelists and the nature of the decision problem.

### 2.3 Bayesian estimation

Bayesian updating estimation is a statistical method used to update prior probability estimates based on new evidence. It is based on Bayes' theorem, which combines prior probabilities with new observed data to calculate posterior probabilities. For discrete variables, the form of the generalized Bayesian's equation is as follows [34]:

(2) $$f\left(A_{i}|E\right)=\frac{\mathrm{Pr}\left(A_{i}\right)\cdot \mathrm{Pr}\left(E|A_{i}\right)}{\sum_{i=1}^{n}\mathrm{Pr}\left(A_{i}\right)\cdot \mathrm{Pr}\left(E|A_{i}\right)}$$

where *f* (*A*_*i*_|*E*) denotes the posterior probability given event *E*, which is updated probability of event *A*_*i*_; Pr (*A*_*i*_) denotes prior probability of event *A*_*i*_; Pr (*E*|*A*_*i*_) denotes relative likelihood, which is based on evidential observations. For continuous variables, the generalized Bayesian's equation is [34]:

(3) $$f\left(\lambda |t\right)=\frac{h\left(\lambda \right)\cdot l\left(t|\lambda \right)}{\int_{-\infty }^{\infty }h\left(\lambda \right)\cdot l\left(t|\lambda \right)d\lambda }$$

where *f* (*λ*|*t*) is the posterior probability density function (pdf) of *λ*; *h*(*λ*) is continuous prior pdf of *λ*; *l* (*t*|*λ*) is the likelihood function based on sample data *t*.

Bayesian updating estimation is widely used in various fields such as machine learning, data analysis, and artificial intelligence [[35], [36], [37]]. It allows for continuous updating of probability estimates based on new data, resulting in more accurate and reliable estimation.

## 3 Developed methodology

**Fig. 3:**
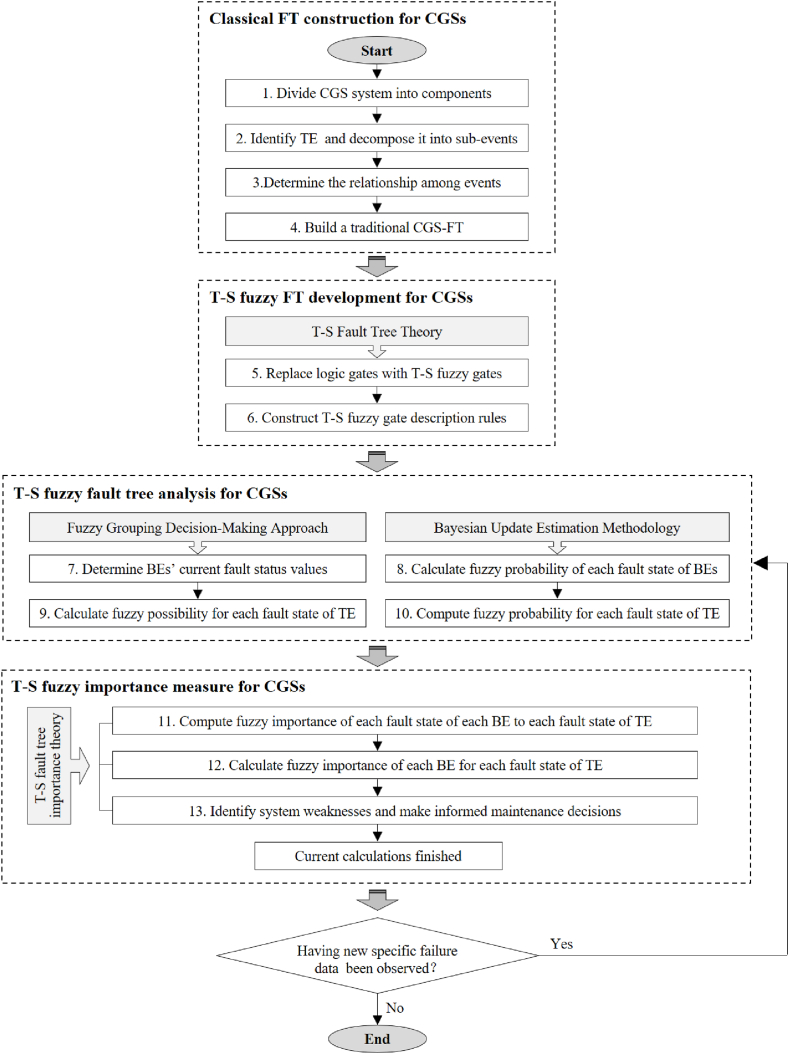
The workflow of the developed methodology for fuzzy reliability assessment of CGS.

To enhance the effectiveness and accuracy of safety management in CGSs, a novel fuzzy reliability assessment methodology has been developed. This methodology expands upon the T-S fault tree [17] and aims to precisely forecast the probability of system failures while simultaneously assisting in identifying weak points. Fig. 3 provides an overall workflow of the approach, which consists of four crucial stages: FT construction for CGSs, T-S fuzzy FT development, T-S fuzzy FTA, and T-S fuzzy importance measure [19]. Detailed algorithms pertaining to these stages are presented in the following sections 3.1, 3.2.

According to this workflow, when new failure data is observed, it is necessary to reassess the fault states, failure probabilities, and calculate the reliability metrics for the top event. By dynamically updating the failure probabilities and importance measure results, engineers and analysts can ensure that the system's performance is continuously monitored and optimized.

### 3.1 T-S fuzzy FT algorithm

#### 3.1.1 Estimation of current fault state values of BEs

Reliability data for BEs or components, such as fault state value, failure rate, and failure probability, form the fundamental basis for evaluating the reliability of the process system. The fault state values for BEs or components can generally be determined from measured parameters of the system, such as pressure and flow [30]. If the available data is insufficient to support this estimation, a fuzzy group decision-making method, which incorporates the knowledge and experience of experts, is proposed to estimate the current fault state value of each BE. The calculation steps can be summarized as follows:

Step 1 Assess the current fault state value of each BE using 7-level language constants such as “Very low, Low, Mildly low, Medium, Mildly high, High, and Very high”.

**Fig. 4:**
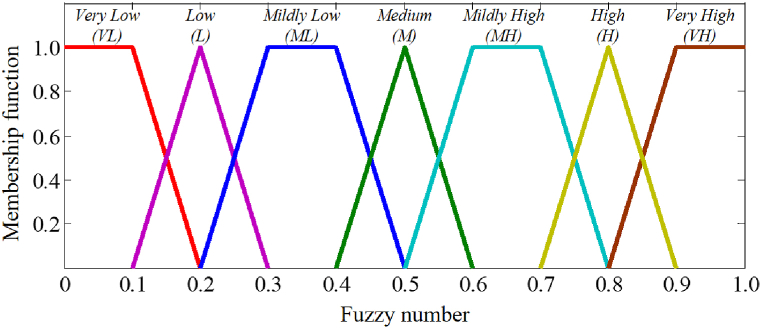
Conversion between linguistic values and fuzzy numbers.

Step 2 Convert experts' language assessments into fuzzy numbers represented by trapezoidal membership functions using Fig. 4 [12].

Step 3 Combine the experts' opinions into a single fuzzy number employing the consistency aggregation method [32], as illustrated in Equations (4), (5).

(4) $${\tilde{p}}_{i}=\sum_{j=1}^{m}w_{j}⊗{\tilde{p}}_{ji}$$

(5) $$w_{j}=\alpha \cdot EID_{j}+\left(1-\alpha \right)\cdot RAD_{ji}$$

where ${\tilde{p}}_{i}$ denotes the synthesized fuzzy number of the *i*th BE's fault state; ${\tilde{p}}_{ji}$ denotes the language judgment of the *j*th expert on the *i*th BE's fault state; *m* represents the number of experts; *w*_*j*_ denotes the comprehensive weighting factor of the *j*th expert; *RAD*_*ji*_ denotes the relative agreement degree of the *j*th expert on the *i*th BE's judgement opinion, and the detailed calculation method can be found in our previous research literature [12]; *EID*_*j*_ denotes the importance degree of the *j*th expert, which can be determined using the Delphi method as presented in Ref. [11]; *α* (0 ≤ *α ≤* 1) is a relaxation factor, which shows the importance *EID*_*j*_ over *RAD*_*ji*_.

Step 4 Employ the center of area defuzzification method, as outlined in Equation (6) [38], to transform the synthesized fuzzy number into a precise fault state value.

(6) $$p_{i}^{*}=\frac{1}{3}\cdot \frac{{\left(p_{4}+p_{3}\right)}^{2}-p_{4}p_{3}-{\left(p_{1}+p_{2}\right)}^{2}+p_{1}p_{2}}{p_{4}+p_{3}-p_{2}-p_{1}}$$

where $p_{i}^{*}$ denotes the crisp fault state value of the *i*th BE; $p_{j}$ denotes the bounds of synthesized fuzzy number.

#### 3.1.2 Estimation of occurrence probabilities for each fault state of BEs

Obtaining precise values for failure rates or failure probabilities for various fault states of each BE (i.e., component, equipment) can be challenging. In general, these probability data are uncertain due to factors such as limited historical failure data, incomplete information, poor data quality, imperfect explanation of failure mechanisms, and changes in system environment [34]. To address this issue, this paper introduces a Bayesian estimation-based method that integrates reliability data from generic databases (such as OREDA, EIReDA, etc.) with continuously collected failure sample data from the site [39]. This approach aims to effectively handle the problem of data uncertainty and achieve accurate estimation of the occurrence probability for each fault state of BEs.

**Fig. 5:**
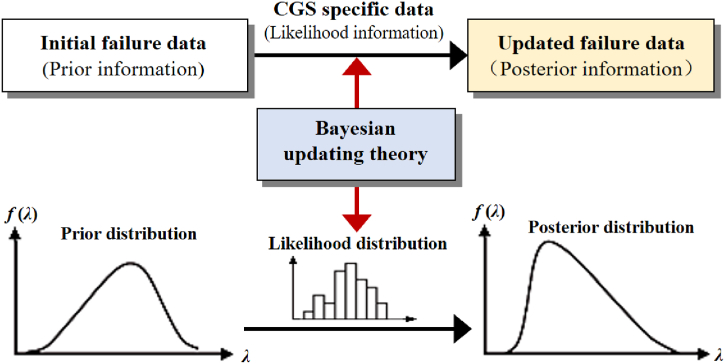
The schematic of Bayesian updating estimation.

The basic concept of Bayesian update estimation is illustrated in Fig. 5. The detailed models for Bayesian update estimation of operational failure rates or demand failure probabilities of system components under various prior distributions are summarized in Table 2. The updated failure rates can capture both the statistical properties of generic failure data and specific characteristics of plant-specific equipment. As the number of failure samples increases, the posterior probabilities can be updated more extensively, leading to a gradual decrease in the uncertainty of the estimation results.

**Table 2:** Bayesian update estimation for running failure rates and demand failure probabilities of BEs.

Failure rate type	Generic data sources-based	Prior distribution		Likelihood function		Posterior distribution	
		Type	Formula	Type	Formula	Type	Formula
Running failure rate (*λ*)	OREDA\ EIReDA	Gamma	$f\left(\lambda \right)=\frac{\beta^{\alpha }}{\Gamma \left(\alpha \right)}\lambda^{\alpha -1}e^{-\beta \lambda }$; $\Gamma \left(\alpha \right)=\int_{0}^{\infty }u^{\alpha -1}e^{-u}du$ *α* = [*E*(*λ*)]^2^/*V*(*λ*), *β* = *E*(*λ*)/*V*(*λ*)	Poisson	$P\left(X=k|\lambda \right)=\frac{{\left(\lambda t\right)}^{k}}{k!}e^{-\lambda t}$ $\hat{\lambda }=k/\tau$	Gamma	$f^{*}\left(\lambda \right)\propto \lambda^{\left(\alpha +k\right)-1}e^{-\left(\beta +\tau \right)\lambda }$ $E^{*}\left(\lambda \right)=\left(\alpha +k\right)/\left(\beta +t\right)$ $\lambda_{0.05}^{*}=\chi_{0.05}^{2}\left(2\alpha +2k\right)/2\left(\beta +\tau \right)$ $\lambda_{0.95}^{*}=\chi_{0.95}^{2}\left(2\alpha +2k\right)/2\left(\beta +\tau \right)$
	CCPS\ EXIDA	Lognormal	$f\left(\lambda \right)=e^{\left[-{\left(\ln\;\lambda -\mu \right)}^{2}/\left(2\sigma^{2}\right)\right]}/\left(\lambda \sigma \sqrt{2\pi }\right)$ Equivalent gamma distribution parameters: $\alpha =\frac{1}{e^{{\left[\ln\;E_{f}\left(\lambda \right)/1.645\right]}^{2}}-1}$, $\beta =\frac{1}{E\left(\lambda \right)\cdot \left\{e^{{\left[\ln\;E_{f}\left(\lambda \right)/1.645\right]}^{2}}-1\right\}}$ *E*_f_ (*λ*) = (*λ*_0.95_/*λ*_0.05_)^1/2^	Poisson	Idem	Gamma	Idem
	–	Jeffreys non-informative	*G*_*a*_ (*α*, *β*) = *G*_*a*_ (0.5, 0) *α* = 0.5; *β* = 0	Poisson	Idem	Gamma	$E_{J}^{*}\left(\lambda \right)=\left(0.5+k\right)/\tau$ $\lambda_{J,0.05}^{*}=\chi_{0.05}^{2}\left(1+2k\right)/2\tau$, $\lambda_{J,0.95}^{*}=\chi_{0.95}^{2}\left(1+2k\right)/2\tau$
Demand failure Probability (*p*)	EIReDA	Beta	$f\left(p\right)=\frac{\Gamma \left(\alpha +\beta \right)}{\Gamma \left(\alpha \right)\Gamma \left(\beta \right)}p^{\alpha -1}{\left(1-p\right)}^{\beta -1}$ $\alpha =\frac{{\left[E\left(p\right)\right]}^{2}\left[1-E\left(p\right)\right]}{V\left(p\right)}-E\left(p\right)$, $\beta =\frac{E\left(p\right){\left[1-E\left(p\right)\right]}^{2}}{V\left(p\right)}+E\left(p\right)-1$	Binomial	$P\left(X=k|p\right)=\frac{n!}{k!\left(n-k\right)!}p^{k}{\left(1-p\right)}^{n-k}$$n=2k/\left(\lambda T_{\text{test}}\right)=2kM_{\text{TBF}}/T_{\text{test}}$	Beta	$f^{*}\left(p\right)\propto p^{\left(\alpha +k\right)-1}{\left(1-p\right)}^{\left(\beta +n-k\right)-1}$ $E^{*}\left(p\right)=\left(\alpha +k\right)/\left(\alpha +\beta +n\right)$$p_{0.05}^{*}=\chi_{0.05}^{2}\left(2\alpha +2k\right)/\left[\left(2\beta +2n-2k\right)+\chi_{0.05}^{2}\left(2\alpha +2k\right)\right]$ $p_{0.95}^{*}=\chi_{0.95}^{2}\left(2\alpha +2k\right)/\left[\left(2\beta +2n-2k\right)+\chi_{0.95}^{2}\left(2\alpha +2k\right)\right]$
	OREDA	Gamma	Equivalent conversion: Gamma prior data→Beta prior parameter→*α*, *β* (*λ*_0.05_, *λ*_mean_, *λ*_0.95_) (*p*_0.05_, *p*_mean_, *p*_0.95_)	Binomial	Idem	Beta	Idem
	CCPS\ EXIDA	Lognormal	$f\left(\lambda \right)=e^{\left[-{\left(\ln\;\lambda -\mu \right)}^{2}/\left(2\sigma^{2}\right)\right]}/\left(\lambda \sigma \sqrt{2\pi }\right)$ Equivalent beta distribution parameters: $\alpha =\frac{1-E\left(p\right)}{e^{{\left[\ln\;E_{f}\left(p\right)/1.645\right]}^{2}}-1}-E\left(p\right)$, $\beta =\frac{{\left[1-E\left(p\right)\right]}^{2}}{\left\{e^{{\left[\ln\;E_{f}\left(p\right)/1.645\right]}^{2}}-1\right\}E\left(p\right)}+E\left(p\right)-1$ *E*_f_ (*p*) = (*p*_0.95_/*p*_0.05_)^1/2^	Binomial	Idem	Beta	Idem
	–	Jeffreys non-informative	*B*_*e*_ (*α*, *β*) = *B*_*e*_ (0.5, 0.5) *α* = 0.5, *β* = 0.5	Binomial	Idem	Beta	$E_{J}^{*}\left(p\right)=\left(k+0.5\right)/\left(n+1\right)$$p_{J,0.05}^{*}=\chi_{0.05}^{2}\left(2k+1\right)/\left[\left(2n-2k+1\right)+\chi_{0.05}^{2}\left(2k+1\right)\right]$ $p_{J,0.95}^{*}=\chi_{0.95}^{2}\left(2k+1\right)/\left[\left(2n-2k+1\right)+\chi_{0.95}^{2}\left(2k+1\right)\right]$

**Notes:***f*(*λ*), *f*(*p*)—Probability density functions; *α*, *β*—distribution parameters; *λ*_mean_, *p*_mean_, *E*(*λ*), *E*(*p*)—Mean values; *V*(*λ*), *V*(*p*)—Variances; $\hat{\lambda }$—Maximum likelihood estimation; *k*—Cumulative running time (hour).

*τ*—Accumulated running time (hour); $\chi_{0.05}^{2}$, $\chi_{0.95}^{2}$—0.05 and 0.95 percentiles of chi-square distribution; *μ*—Mean value of ln*λ*; *σ*—Standard deviation of ln*λ*; *E*_f_(*λ*), *E*_f_(*p*)—Error factors; *n*—Number of demands.

*T*_test_—Proof test interval (hour); *M*_TBF_—Mean time between failures (hour); *—Identifier of posterior data.

#### 3.1.3 Calculation of occurrence probabilities for each fault state of top event

If the reliability data obtained (such as failure probabilities, fuzzy probabilities, etc.) of each fault state $S_{i}^{\left(a_{i}\right)}\;\left(a_{i}=1,2,\cdots ,k_{i}\right)$ of the lower-level events $x_{i}\;\left(i=1,2,\cdots ,n\right)$ in the T-S fuzzy gate are represented by $P_{\left(l\right)}\left(x_{i}=S_{i}^{\left(a_{i}\right)}\right)\;\left(a_{i}=1,2,\cdots ,k_{i}\right)$, then the reliability data $P\left(y=S_{y}^{\left(b_{y}\right)}\right)$ for each fault state $S_{y}^{\left(b_{y}\right)}\;\left(b_{y}=1,2,\cdots ,k_{y}\right)$ of the upper-level event *y* can be expressed as [25]:

(7) $$P\left(y=S_{y}^{\left(b_{y}\right)}\right)=\sum_{l=1}^{r}P_{\left(l\right)}^{*}P_{\left(l\right)}\left(y=S_{y}^{\left(b_{y}\right)}\right)$$

(8) $$P_{\left(l\right)}^{*}=\prod_{i=1}^{n}P_{\left(l\right)}\left(x_{i}=S_{i}^{\left(a_{i}\right)}\right)$$

where $P_{\left(l\right)}^{*}$ is the execution degree of the fuzzy rule *l*.

According to the established rule, the sum of the reliability data for each fault state of the upper-level event *y*, $\sum_{b_{y}=1}^{k_{y}}P\left(y=S_{y}^{\left(b_{y}\right)}\right)$, should be equal to 1. By following the structure of the T-S FFT of the target CGS, the reliability data $P\left(T=T^{\left(q\right)}\right)$ for each fault state $T^{\left(q\right)}\left(q=1,2,\cdots ,k_{q}\right)$ of the top event (*T*) can be derived through a step-wise upward solving process using Equations (7) and (8).

#### 3.1.4 Calculation of fuzzy possibilities for each fault state of top event

If the current fault states of the lower-level events $x_{i}\;\left(i=1,2,\cdots ,n\right)$ of the T-S fuzzy gate are ${\hat{x}}_{i}\;\left(i=1,2,\cdots ,n\right)$, the fuzzy possibility $p\left(y=S_{y}^{\left(b_{y}\right)}\right)$ of each fault state $S_{y}^{\left(b_{y}\right)}\;\left(b_{y}=1,2,\cdots ,k_{y}\right)$ of the upper-level event *y* can be estimated based on the T-S model as follows [17].

(9) $$p\left(y=S_{y}^{\left(b_{y}\right)}\right)=\sum_{l=1}^{r}\beta_{\left(l\right)}^{*}\left(\hat{x}\right)P_{\left(l\right)}\left(y=S_{y}^{\left(b_{y}\right)}\right)$$

(10) $$\beta_{\left(l\right)}^{*}\left(\hat{x}\right)=\prod_{i=1}^{n}\mu_{F\left(l\right)}\left({\hat{x}}_{i}\right)/\sum_{l=1}^{r}\prod_{i=1}^{n}\mu_{F\left(l\right)}\left({\hat{x}}_{i}\right)$$

where $\beta_{\left(l\right)}^{*}\left(\hat{x}\right)$ is the normalized execution degree of the input rule *l* (*l* = 1, 2, …, *r*) of the T-S fuzzy gate; $\mu_{F\left(l\right)}\left({\hat{x}}_{i}\right)$ is the membership degree of the corresponding fuzzy set of ${\hat{x}}_{i}$ in rule *l*, and the sum of the membership degrees of the current fault state values of lower-level events to each fault state is 1.

According to the structure of the T-S FFT of the target CGS, the fuzzy possibility $p\left(T=T^{\left(q\right)}\right)$ for each fault state $T^{\left(q\right)}\left(q=1,2,\cdots ,k_{q}\right)$ of the top event *T* can be obtained by solving upward step by step using Equations (9), (10).

### 3.2 T-S fuzzy importance measure

The occurrence of BEs will increase the fuzzy probability of the top event, and the importance degree reflects the influence of BEs (i.e., system components) on the reliability of the entire FT (i.e., the process system). The importance measure can be used to identify weak links in the system, improve design, and optimize reliability. According to the T-S importance theory [19], the fuzzy probability of BE *x*_*i*_ with fault state $S_{i}^{\left(a_{i}\right)}$ is $\tilde{P}\left(x_{i}=S_{i}^{\left(a_{i}\right)}\right)$, and its T-S fuzzy probability importance $I_{F_{u}}^{\left(T_{q}\right)}\left(x=S_{i}^{\left(a_{i}\right)}\right)$ for top event *T* with fault state *T*_*q*_ is calculated as follows:

(11) $$I_{F_{u}}^{\left(T_{q}\right)}\left(x_{i}=S_{i}^{\left(a_{i}\right)}\right)=E\left[P\left(T_{q},\tilde{P}\left(x_{i}=S_{i}^{\left(a_{i}\right)}\right)=1\right)-P\left(T_{q},\tilde{P}\left(x_{i}=S_{i}^{\left(a_{i}\right)}\right)=0\right)\right]$$

where $P\left(T_{q},\tilde{P}\left(x=S_{i}^{\left(a_{i}\right)}\right)=1\right)$ denotes the fuzzy probability of the top event *T* in fault state *T*_*q*_ when $\tilde{P}\left(x_{i}=S_{i}^{\left(a_{i}\right)}\right)=1$; $P\left(T_{q},\tilde{P}\left(x=S_{i}^{\left(a_{i}\right)}\right)=0\right)$ denotes the fuzzy probability of the top event *T* in fault state *T*_*q*_ when $\tilde{P}\left(x_{i}=S_{i}^{\left(a_{i}\right)}\right)=0$; $E\left[\tilde{A}\right]$ represents the center value of the fuzzy subset $\tilde{A}$, which converts the fuzzy subset into a precise value.

The calculation of the T-S fuzzy importance $I_{F_{u}}^{\left(T_{q}\right)}\left(x=S_{i}^{\left(a_{i}\right)}\right)$ of the BE *x*_*i*_ to the top event *T* in fault state *T*_*q*_ can be determined as follows [19]. The greater the fuzzy importance of a BE, the weaker the link where the BE is situated.

(12) $$I_{F_{u}}^{\left(T_{q}\right)}\left(x_{i}\right)=\frac{\sum_{a_{i}=2}^{k_{i}}I_{F_{u}}^{\left(T_{q}\right)}\left(x_{i}=S_{i}^{\left(a_{i}\right)}\right)}{k_{i}-1}$$

where *k*_*i*_ denotes the number of fault states of the BE *x*_*i*_.

## 4 A case study

To demonstrate the effectiveness of the proposed methodology in the reliability assessment of CGSs, a city gas distribution station (CGDS) is examined as a case study. The CGDS performs several primary functions, including impurity separation, gas distribution, pigging and safety barrier. The CGDS comprises five critical units with multiple components, each of which has the potential to lead to the failure of the station system, as outlined in Table 3.

**Table 3:** Units and components of the CGDS.

Units	Components	Functional description
Separation	Filter separator (*x*_1_)	Removing solid and liquid impurities carried in the gas
	Drain valve (*x*_2_)	Draining impurities accumulated in the separator
	Cut-off ball valve (*x*_3_)	Isolating the inlet and outlet of the separator
Distribution	Orifice flowmeter (*x*_4_)	Measuring the gas volume flow rate assigned to the user
	Gas rectifier (*x*_5_)	Stabilizing gas flow to ensure metering accuracy
	Process manifold (*x*_6_)	Collecting, balancing and distributing gases
	Pressure regulator (*x*_7_)	Controlling gas supply pressure and flow
Pigging	Pig transceiver (*x*_8_)	Sending and receiving pig
	Pig pass indicator (*x*_9_)	Detecting the passage of pig
Safety barrier	Emergency shut-off valve (*x*_10_)	Cutting off the gas supply to avoid accidents
	Safety valve (*x*_11_)	Draining excess pressure from process pipework and equipment
	Vent valve (*x*_12_)	Venting pressurized gases in non-operational or emergency situations
	Check valve (*x*_13_)	Blocking gas backflow

### 4.1 Constructing the T-S fuzzy fault tree of the CGDS system

**Fig. 6:**
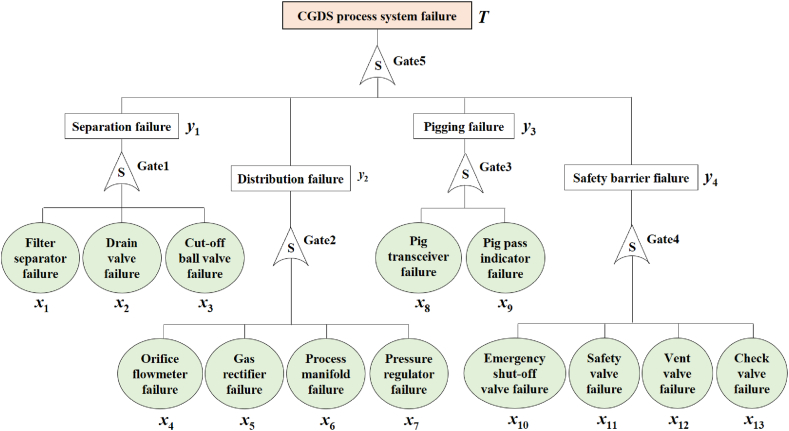
The developed T-S fuzzy fault tree of CGDS system.

The developed T-S FFT with CGDS system failure (*T*) as the top event is depicted in Fig. 6. Gates 1 to 5 represent the T-S fuzzy gates, as explained previously. The occurrence of faults in one or more components determines the likelihood of CGDS system failure, depending on the fault states of the components and whether these failures are critical or not. Three fault states (0, 0.5, 1) are defined for events *x*_1_∼*x*_3_, *x*_7_∼*x*_8_, *x*_10_, *x*_12_ and *y*_1_∼*y*_4_, with their membership function parameters set as *s*_L_ = *s*_R_ = 0.1 and *m*_L_ = *m*_R_ = 0.3. Furthermore, two fault states (0,1) are defined for events *x*_4_∼*x*_6_, *x*_9_, *x*_11_, *x*_13_, with the membership function parameters set as *s*_L_ = *s*_R_ = 0.25 and *m*_L_ = *m*_R_ = 0.5. The description rules for T-S fuzzy gates 1 to 5 are obtained based on historical failure data and experts’ experience, as shown in Table 4, Table 5, Table 6, Table 7, Table 8, respectively.

**Table 4:** The description rules of T-S fuzzy gate 1.

Rule	*x*_1_	*x*_2_	*x*_3_	*y*_1_		
				0	0.5	1
1	0	0	0	1	0	0
2	0	0	0.5	0.2	0.4	0.4
3	0	0	1	0	0	1
4	0	0.5	0	0.2	0.4	0.4
5	0	0.5	0.5	0.1	0.3	0.6
6	0	0.5	1	0	0	1
7	0	1	0	0	0	1
8	0	1	0.5	0	0	1
9	0	1	1	0	0	1
…	…	…	…	…	…	…
27	1	1	1	0	0	1

**Table 5:** The description rules of T-S fuzzy gate 2.

Rule	*x*_4_	*x*_5_	*x*_6_	*x*_7_	*y*_2_		
					0	0.5	1
1	0	0	0	0	1	0	0
2	0	0	0	0.5	0.2	0.3	0.5
3	0	0	0	1	0	0	1
4	0	0	1	0	0.2	0.2	0.6
5	0	0	1	0.5	0.1	0.1	0.8
6	0	0	1	1	0	0	1
7	0	1	0	0	0.4	0.4	0.2
8	0	1	0	0.5	0.2	0.2	0.6
9	0	1	0	1	0	0	1
…	…	…	…	…	…	…	…
24	1	1	1	1	0	0	1

**Table 6:** The description rules of T-S fuzzy gate 3.

Rule	*x*_8_	*x*_9_	*y*_3_		
			0	0.5	1
1	0	0	1	0	0
2	0	1	0	0	1
3	0.5	0	0.1	0.4	0.5
4	0.5	1	0	0	1
5	1	0	0	0	1
6	1	1	0	0	1

**Table 7:** The description rules of T-S fuzzy gate 4.

Rule	*x*_10_	*x*_11_	*x*_12_	*x*_13_	*y*_4_		
					0	0.5	1
1	0	0	0	0	1	0	0
2	0	0	0	1	0.7	0.2	0.1
3	0	0	0.5	0	0.5	0.1	0.4
4	0	0	0.5	1	0.1	0.3	0.6
5	0	0	1	0	0.1	0.2	0.7
6	0	0	1	1	0.1	0.1	0.8
7	0	1	0	0	0	0	1
8	0	1	0	1	0	0	1
9	0	1	0.5	0	0	0	1
…	…	…	…	…	…	…	…
36	1	1	1	1	0	0	1

**Table 8:** The description rules of T-S fuzzy gate 5.

Rule	*y*_1_	*y*_2_	*y*_3_	*y*_4_	*T*		
					0	0.5	1
1	0	0	0	0	1	0	0
2	0	0	0	0.5	0.4	0.4	0.2
3	0	0	0	1	0	0	1
4	0	0	0.5	0	0.8	0.1	0.1
5	0	0	0.5	0.5	0.3	0.4	0.3
6	0	0	0.5	1	0	0	1
7	0	0	1	0	0	0	1
8	0	0	1	0.5	0	0	1
9	0	0	1	1	0	0	1
…	…	…	…	…	…	…	…
81	1	1	1	1	0	0	1

### 4.2 Estimating the current fault state values and failure rates of the CGDS's components

To assess the current fault state value of each component in the CGDS system, a fuzzy grouping decision-making method is proposed here. A team of five experts, with extensive experience in field production and management, is assembled for this purpose. Each expert evaluates the current fault state of each component using the 7-level language constants depicted in Fig. 4. These language values are then transformed into fuzzy numbers, represented by trapezoidal membership functions. The fuzzy numbers of the experts' opinions are subsequently synthesized into a unified result using a consistency aggregation method [12]. Finally, a single crisp value for the fault state of each component is obtained by applying the center of area defuzzification technique [38]. The estimation results of the fault state values for each component are presented in column of Table 9, which will serve as the base data for the fuzzy possibility calculations of the CGDS fault states (see Table 10).

**Table 9:** The estimation results of current fault state values and of each component.

Component	Fault state value by fuzzy group decision-making			Fault probability by Bayesian models	
	Expert's Linguistic judgement	Synthesized fuzzy number	Crisp fault State value	Posterior failure rate (lower, mean, upper) (10^−6^)	
				Fault state = 1.0	Fault state = 0.5
*x*_1_	*(L, L, ML, VL, L)*	(0.12, 0.21, 0.25, 0.35)	0.2	(9.85, 41.57, 75.86)	(54.00, 136.30, 232.29)
*x*_2_	*(L, VL, M, L, LM)*	(0.25, 0.34, 0.35, 0.45)	0.3	(5.32, 35.03, 70.94)	(15.50, 54.03, 100.62)
*x*_3_	*(VL, VL, L, VL, VL)*	(0.03, 0.06, 0.13, 0.23)	0.1	(2.90, 8.48, 16.44)	(10.55, 39.50, 81.24)
*x*_4_	*(L, VL, L, L, VL)*	(0.07, 0.14, 0.17, 0.27)	0.2	(7.79, 34.01, 75.31)	(7.79, 34.01, 75.31)
*x*_5_	*(L, VL, M, M, VL)*	(0.24, 0.32, 0.34, 0.44)	0.3	(0.30, 1.43, 2.85)	(0.30, 1.43, 2.85)
*x*_6_	*(VL, VL, VL, VL, L)*	(0.03, 0.06, 0.13, 0.23)	0.1	(1.62, 9.42, 21.64)	(1.62, 9.42, 21.64)
*x*_7_	*(VL, L, VL, L, L)*	(0.07, 0.14, 0.17, 0.27)	0.2	(2.39, 20.41, 53.16)	(14.74, 47.62, 95.71)
*x*_8_	*(L, L, M, ML, ML)*	(0.22, 0.32, 0.36, 0.46)	0.3	(2.16, 18.37, 47.85)	(7.01, 30.61, 67.78)
*x*_9_	*(ML, L, VL, ML, VL)*	(0.13, 0.21, 0.29, 0.39)	0.3	(2.69, 22.96, 59.81)	(2.69, 22.96, 59.81)
*x*_10_	*(VL, L, VL, VL, L)*	(0.05, 0.11, 0.15, 0.25)	0.1	(0.53, 5.22, 10.78)	(0.52, 3.43, 6.53)
*x*_11_	*(VL, L, VL, ML, L)*	(0.10, 0.17, 0.23, 0.33)	0.2	(0.55, 5.87, 12.27)	(0.55, 5.87, 12.27)
*x*_12_	*(VL, L, ML, ML, L)*	(0.13, 0.22, 0.28, 0.38)	0.3	(2.43, 13.65, 32.37)	(5.58, 20.47, 42.96)
*x*_13_	*(L, VL, L, VL, VL)*	(0.06, 0.11, 0.16, 0.26)	0.1	(0.30, 8.59, 17.22)	(0.30, 8.59, 17.22)

**Table 10:** The estimation results of the fuzzy possibility of occurrence of each fault state for *y*_1_.

Rule	$\mu_{F\left(l\right)}\left({\hat{x}}_{1}\right)$	$\mu_{F\left(l\right)}\left({\hat{x}}_{2}\right)$	$\mu_{F\left(l\right)}\left({\hat{x}}_{3}\right)$	$\beta_{\left(l\right)}^{*}\left(\hat{x}\right)$	$\beta_{\left(l\right)}^{*}\left(\hat{x}\right)\cdot P_{\left(l\right)}\left(y_{1}\right)$		
					$y_{1}=0$	$y_{1}=0.5$	$y_{1}=1$
1	4/7	1/6	1	0.094	0.094	0	0
2	4/7	1/6	0	0.006	0.001	0.002	0.002
4	4/7	5/6	1	0.435	0.087	0.174	0.174
5	4/7	5/6	0	0.028	0.003	0.008	0.017
10	3/7	1/6	1	0.073	0.015	0.022	0.037
11	3/7	1/6	0	0.005	0	0.001	0.003
13	3/7	5/6	1	0.337	0.034	0.067	0.236
14	3/7	5/6	0	0.021	0	0.004	0.017
$p\left(y_{1}\right)=\sum \beta_{\left(l\right)}^{*}\left(\hat{x}\right)\cdot P_{\left(l\right)}\left(y_{1}\right)$					**0.234**	**0.279**	**0.486**

Due to the limited availability of failure record data, the Bayesian estimation models presented in Table 2 are employed to estimate the failure probabilities of components in the CGDS system, in conjunction with generic data sources [40]. The results of the probability estimation are provided in columns 5 to 6 of Table 9. Considering the uncertainties in the failure rate parameters of the system components, the failure rate values are treated as fuzzy probabilities. For example, the failure probabilities of component *x*_1_ with a failure state of 1 are 9.85 × 10^−6^, 4.16 × 10^−5^, 7.60 × 10^−5^, while the fuzzy probabilities of its failure state of 0.5 are 5.40 × 10^−5^, 1.36 × 10^−4^, 2.32 × 10^−4^.

### 4.3 Calculating the occurrence probabilities of each fault state of the CGDS system

According to Table 4, Table 5, Table 6, Table 7 and Equations (7) and (8), the occurrence probabilities of *y*_1_ to *y*_4_ with fault states of 0, 0.5, and 1 can be calculated as follows.

$$P\left(y_{1}=1.0\right)=\sum_{l=1}^{27}P_{\left(l\right)}^{*}P_{\left(l\right)}\left(y_{1}=1.0\right)=\left(5.55,19.06,35.21\right)\times 10^{-5}$$

$$P\left(y_{1}=0.5\right)=\sum_{l=1}^{27}P_{\left(l\right)}^{*}P_{\left(l\right)}\left(y_{1}=0.5\right)=\left(2.66,7.83,14.24\right)\times 10^{-5}$$

$$P\left(y_{2}=1.0\right)=\sum_{l=1}^{24}P_{\left(l\right)}^{*}P_{\left(l\right)}\left(y_{2}=1.0\right)=\left(1.86,8.42,18.99\right)\times 10^{-5}$$

$$P\left(y_{2}=0.5\right)=\sum_{l=1}^{24}P_{\left(l\right)}^{*}P_{\left(l\right)}\left(y_{2}=0.5\right)=\left(0.49,1.67,3.42\right)\times 10^{-5}$$

$$P\left(y_{3}=1.0\right)=\sum_{l=1}^{6}P_{\left(l\right)}^{*}P_{\left(l\right)}\left(y_{3}=1.0\right)=\left(0.84,5.66,14.15\right)\times 10^{-5}$$

$$P\left(y_{3}=0.5\right)=\sum_{l=1}^{6}P_{\left(l\right)}^{*}P_{\left(l\right)}\left(y_{3}=0.5\right)=\left(0.28,1.23,2.71\right)\times 10^{-5}$$

$$P\left(y_{4}=1.0\right)=\sum_{l=1}^{36}P_{\left(l\right)}^{*}P_{\left(l\right)}\left(y_{4}=1.0\right)=\left(0.53,3.11,6.72\right)\times 10^{-5}$$

$$P\left(y_{4}=0.5\right)=\sum_{l=1}^{36}P_{\left(l\right)}^{*}P_{\left(l\right)}\left(y_{4}=0.5\right)=\left(0.12,0.68,1.49\right)\times 10^{-5}$$

According to Table 8 and Equations (7) and (8) and based on the above results, the occurrence probabilities of the CGDS system (*T*) with fault states 0, 0.5 and 1 are calculated as:

$$P\left(T=1.0\right)=\sum_{l=1}^{81}P_{\left(l\right)}^{*}P_{\left(l\right)}\left(T=1.0\right)=\left(7.91,31.51,63.63\right)\times 10^{-5}$$

$$P\left(T=0.5\right)=\sum_{l=1}^{81}P_{\left(l\right)}^{*}P_{\left(l\right)}\left(T=0.5\right)=\left(1.90,7.29,15.31\right)\times 10^{-5}$$

P(T=0)=1−P(T=1.0)−P(T=0.5)=(0.99990,0.99961,0.99921)

The findings suggest that the failure probability of the CGDS system is on the same order of magnitude as that of each individual component. Additionally, the probability of a fault state 1 occurring in each unit (impurity separation, gas distribution, pigging, and safety barrier) is lower than that of the CGDS system under the same fault state.

### 4.4 Calculating the fuzzy possibilities of each fault state of the CGDS system

Based on the current fault state value of each component (refer to column 4 of Table 9) and Equation (1), the membership degrees ($\mu_{F\left(l\right)}\left({\hat{x}}_{i}\right)$) of the fault states of the components (*x*_1_∼*x*_13_) in each rule in Table 4, Table 5, Table 6, Table 7 are calculated. Subsequently, the execution degree ($\beta_{\left(l\right)}^{*}\left(\hat{x}\right)$) of each rule is determined using Equation (10). Following this, the fuzzy possibilities ($p\left(y_{i}\right)$) with the fault states of 0, 0.5 and 1 for each unit (*y*_1_∼*y*_4_) are derived based on Equation (9). The resulting data is presented in Table 10, Table 11, Table 12, Table 13.

**Table 11:** The estimation results of the fuzzy possibility of occurrence of each fault state for *y*_2_.

Rule	$\mu_{F\left(l\right)}\left({\hat{x}}_{4}\right)$	$\mu_{F\left(l\right)}\left({\hat{x}}_{5}\right)$	$\mu_{F\left(l\right)}\left({\hat{x}}_{6}\right)$	$\mu_{F\left(l\right)}\left({\hat{x}}_{7}\right)$	$\beta_{\left(l\right)}^{*}\left(\hat{x}\right)$	$\beta_{\left(l\right)}^{*}\left(\hat{x}\right)\cdot P_{\left(l\right)}\left(y_{2}\right)$		
						$y_{2}=0$	$y_{2}=0.5$	$y_{2}=1$
1	1	5/6	1	7/9	0.637	0.637	0	0
2	1	5/6	1	2/9	0.182	0.036	0.055	0.091
7	1	1/6	1	7/9	0.141	0.056	0.056	0.028
8	1	1/6	1	2/9	0.040	0.008	0.008	0.024
$p\left(y_{2}\right)=\sum \beta_{\left(l\right)}^{*}\left(\hat{x}\right)\cdot P_{\left(l\right)}\left(y_{2}\right)$						**0.738**	**0.119**	**0.143**

**Table 12:** The estimation results of the fuzzy possibility of occurrence of each fault state for *y*_3_.

Rule	$\mu_{F\left(l\right)}\left({\hat{x}}_{8}\right)$	$\mu_{F\left(l\right)}\left({\hat{x}}_{9}\right)$	$\beta_{\left(l\right)}^{*}\left(\hat{x}\right)$	$\beta_{\left(l\right)}^{*}\left(\hat{x}\right)\cdot P_{\left(l\right)}\left(y_{3}\right)$		
				$y_{3}=0$	$y_{3}=0.5$	$y_{3}=1$
1	1/5	1	0.208	0.208	0	0
2	1/5	0	0.002	0	0	0.002
3	4/5	1	0.783	0.078	0.313	0.391
4	4/5	0	0.007	0	0	0
$p\left(y_{3}\right)=\sum \beta_{\left(l\right)}^{*}\left(\hat{x}\right)\cdot P_{\left(l\right)}\left(y_{3}\right)$				**0.286**	**0.313**	**0.401**

**Table 13:** The estimation results of the fuzzy possibility of occurrence of each fault state for *y*_4_.

Rule	$\mu_{F\left(l\right)}\left({\hat{x}}_{10}\right)$	$\mu_{F\left(l\right)}\left({\hat{x}}_{11}\right)$	$\mu_{F\left(l\right)}\left({\hat{x}}_{12}\right)$	$\mu_{F\left(l\right)}\left({\hat{x}}_{13}\right)$	$\beta_{\left(l\right)}^{*}\left(\hat{x}\right)$	$\beta_{\left(l\right)}^{*}\left(\hat{x}\right)\cdot P_{\left(l\right)}\left(y_{4}\right)$		
						$y_{4}=0$	$y_{4}=0.5$	$y_{4}=1$
1	6/7	1	1/2	1	0.409	0.409	0	0
3	6/7	1	1/2	1	0.440	0.220	0.044	0.176
13	1/7	1	1/2	1	0.073	0.036	0.007	0.029
15	1/7	1	1/2	1	0.078	0.031	0.016	0.031
$p\left(y_{4}\right)=\sum \beta_{\left(l\right)}^{*}\left(\hat{x}\right)\cdot P_{\left(l\right)}\left(y_{4}\right)$						**0.697**	**0.067**	**0.236**

Finally, by utilizing the fuzzy possibility ($p\left(y_{i}\right)$) of *y*_*i*_ instead of its membership degree ($\mu_{F\left(l\right)}\left({\hat{y}}_{i}\right)$), and applying Equations (1), (10), (9) successively, the fuzzy possibilities with the fault states of 0, 0.5, and 1 for the CGDS system (*T*) are calculated as follows. The specific results are presented in Table 14. It is evident that simultaneous minor failures in multiple components of the CGDS system increase the probability of a major system failure.

$$p\left(T=0\right)=\sum_{l=1}^{81}\beta_{\left(l\right)}^{*}\left(\hat{y}\right)P_{\left(l\right)}\left(T=0\right)=0.149$$

$$p\left(T=0.5\right)=\sum_{l=1}^{81}\beta_{\left(l\right)}^{*}\left(\hat{y}\right)P_{\left(l\right)}\left(T=0.5\right)=0.096$$

$$p\left(T=1.0\right)=\sum_{l=1}^{81}\beta_{\left(l\right)}^{*}\left(\hat{y}\right)P_{\left(l\right)}\left(T=1.0\right)=0.755$$

**Table 14:** The estimation results of the fuzzy possibility of occurrence of each fault state for *T*.

Rule	$\mu_{F\left(l\right)}\left({\hat{y}}_{1}\right)$	$\mu_{F\left(l\right)}\left({\hat{y}}_{2}\right)$	$\mu_{F\left(l\right)}\left({\hat{y}}_{3}\right)$	$\mu_{F\left(l\right)}\left({\hat{y}}_{4}\right)$	$\beta_{\left(l\right)}^{*}\left(\hat{y}\right)$	$\beta_{\left(l\right)}^{*}\left(\hat{y}\right)\cdot P_{\left(l\right)}\left(T\right)$		
						T=0	T=0.5	T=1
1	0.234	0.738	0.286	0.697	0.034	0.034	0	0
2	0.234	0.738	0.286	0.067	0.003	0.001	0.001	0.001
3	0.234	0.738	0.286	0.236	0.012	0	0	0.012
4	0.234	0.738	0.313	0.697	0.038	0.030	0.004	0.004
5	0.234	0.738	0.313	0.067	0.004	0.001	0.001	0.001
6	0.234	0.738	0.313	0.236	0.013	0	0	0.013
7	0.234	0.738	0.401	0.697	0.048	0	0	0.048
8	0.234	0.738	0.401	0.067	0.005	0	0	0.005
9	0.234	0.738	0.401	0.236	0.016	0	0	0.016
10	0.234	0.119	0.286	0.697	0.006	0.004	0.001	0.001
…	…	…	…	…	…	…	…	…
28	0.279	0.738	0.286	0.697	0.041	0.021	0.012	0.008
29	0.279	0.738	0.286	0.067	0.004	0	0.002	0.002
…	…	…	…	…	…	…	…	…
55	0.486	0.738	0.286	0.697	0.072	0	0	0.072
56	0.486	0.738	0.286	0.067	0.007	0	0	0.007
…	…	…	…	…	…	…	…	…
81	0.486	0.143	0.401	0.236	0.007	0	0	0.007
$p\left(T\right)=\sum \beta_{\left(l\right)}^{*}\left(\hat{y}\right)\cdot P_{\left(l\right)}\left(T\right)$						**0.149**	**0.096**	**0.755**

### 4.5 Evaluating the fuzzy importance of each component of the CGDS system

Replacing the fuzzy probability of component *x*_1_ with a fault state of 0.5 with 1 and 0, respectively, and combining Equations (6) and (7) and (10), the T-S fuzzy importance of the component *x*_1_ with the fault states of 0.5 and 1 for the CGDS system (*T*) with the fault state of 0.5, respectively, are obtained as follows. Similarly, the T-S fuzzy importance of the fault states of 0.5 and 1 for each component to the fault state 0.5 of the CGDS system (*T*) can be obtained as shown in Table 15.

$$I_{F_{u}}^{\left(0.5\right)}\left(x_{i}=0.5\right)=E\left[P\left(0.5,\tilde{P}\left(x_{i}=0.5\right)=1\right)-P\left(0.5,\tilde{P}\left(x_{i}=0.5\right)=0\right)\right]=0.09$$

$$I_{F_{u}}^{\left(0.5\right)}\left(x_{i}=1\right)=E\left[P\left(0.5,\tilde{P}\left(x_{i}=1\right)=1\right)-P\left(0.5,\tilde{P}\left(x_{i}=1\right)=0\right)\right]=0$$

**Table 15:** T-S fuzzy importance of component's fault states 0.5 and 1 for fault state 0.5 of the CGDS system (T).

Fault state of *x*_*i*_	$I_{F_{u}}^{\left(0.5\right)}\left(x_{i}=S_{i}^{\left(a_{i}\right)}\right)$	Fault state of *x*_*i*_	$I_{F_{u}}^{\left(0.5\right)}\left(x_{i}=S_{i}^{\left(a_{i}\right)}\right)$	Fault state of *x*_*i*_	$I_{F_{u}}^{\left(0.5\right)}\left(x_{i}=S_{i}^{\left(a_{i}\right)}\right)$
$x_{1}=0.5$	0.09	$x_{6}=0.5$	–	$x_{11}=0.5$	0
$x_{1}=1$	0	$x_{6}=1$	0.34	$x_{11}=1$	–
$x_{2}=0.5$	0.12	$x_{7}=0.5$	0.31	$x_{12}=0.5$	0.04
$x_{2}=1$	0	$x_{7}=1$	0.50	$x_{12}=1$	0.08
$x_{3}=0.5$	0.12	$x_{8}=0.5$	0.04	$x_{13}=0.5$	–
$x_{3}=1$	0	$x_{8}=1$	0	$x_{13}=1$	0.08
$x_{4}=0.5$	–	$x_{9}=0.5$	–		
$x_{4}=1$	0.50	$x_{9}=1$	0		
$x_{5}=0.5$	–	$x_{10}=0.5$	0.04		
$x_{5}=1$	0.18	$x_{10}=1$	0		

Utilizing the aforementioned method, the T-S fuzzy importance of the fault states of 0.5 and 1 for each component to the fault state 1 of the CGDS system can be derived, as presented in Table 16.

**Table 16:** T-S fuzzy importance of component's fault states 0.5 and 1 for fault state 1 of the CGDS system (T).

Fault state of *x*_*i*_	$I_{F_{u}}^{\left(1\right)}\left(x_{i}=S_{i}^{\left(a_{i}\right)}\right)$	Fault state of *x*_*i*_	$I_{F_{u}}^{\left(1\right)}\left(x_{i}=S_{i}^{\left(a_{i}\right)}\right)$	Fault state of *x*_*i*_	$I_{F_{u}}^{\left(1\right)}\left(x_{i}=S_{i}^{\left(a_{i}\right)}\right)$
$x_{1}=0.5$	0.56	$x_{6}=0.5$	0	$x_{11}=0.5$	0
$x_{1}=1$	1	$x_{6}=1$	0.14	$x_{11}=1$	1
$x_{2}=0.5$	0.48	$x_{7}=0.5$	0.13	$x_{12}=0.5$	0.42
$x_{2}=1$	1	$x_{7}=1$	0.20	$x_{12}=1$	0.74
$x_{3}=0.5$	0.48	$x_{8}=0.5$	0.54	$x_{13}=0.5$	0
$x_{3}=1$	1	$x_{8}=1$	1	$x_{13}=1$	0.14
$x_{4}=0.5$	0	$x_{9}=0.5$	0		
$x_{4}=1$	0.20	$x_{9}=1$	1		
$x_{5}=0.5$	0	$x_{10}=0.5$	0.42		
$x_{5}=1$	0.08	$x_{10}=1$	1		

Next, using Equation (12), the T-S fuzzy importance of the component *x*_1_ with the fault states of 0.5 and 1 for the CGDS system (*T*) can be obtained as follows.

$$I_{F_{u}}^{\left(0.5\right)}\left(x_{1}\right)=\frac{I_{F_{u}}^{\left(0.5\right)}\left(x_{1}=0.5\right)+I_{F_{u}}^{\left(0.5\right)}\left(x_{1}=1\right)}{2}=0.05$$

$$I_{F_{u}}^{\left(1\right)}\left(x_{1}\right)=\frac{I_{F_{u}}^{\left(1\right)}\left(x_{1}=0.5\right)+I_{F_{u}}^{\left(1\right)}\left(x_{1}=1\right)}{2}=0.78$$

Similarly, the T-S fuzzy importance of each component to the CGDS system (*T*) with fault states of 0.5 and 1 can be obtained as shown in Table 17. The results indicate that when the fault state of the system is 0.5, the fuzzy importance ranking of the components is *x*_7_>*x*_4_>*x*_6_>*x*_5_>*x*_2_ (*x*_3_,*x*_12_) > *x*_1_>*x*_13_>*x*_8_ (*x*_10_) > *x*_9_ (*x*_11_). The pressure regulator has the most significant impact on the system, followed by the orifice flowmeter, process manifold and gas rectifier. The pig pass indicator and safety valve have the least impact. When the fault state of the system is 1, the importance ranking of the components is *x*_1_>*x*_8_>*x*_2_ (*x*_3_) > *x*_10_>*x*_12_>*x*_9_ (*x*_11_) > *x*_7_>*x*_4_>*x*_6_ (*x*_13_) > *x*_5_. The filter separator has the greatest impact on the system, followed by the pig transceiver, drain valve, cut-off ball valve and emergency shut-off valve.

**Table 17:** T-S fuzzy importance of components.

Component	$I_{F_{u}}^{\left(0.5\right)}\left(x_{i}\right)$	$I_{F_{u}}^{\left(1\right)}\left(x_{i}\right)$	Component	$I_{F_{u}}^{\left(0.5\right)}\left(x_{i}\right)$	$I_{F_{u}}^{\left(1\right)}\left(x_{i}\right)$
*x*_1_	0.05	0.78	*x*_8_	0.02	0.77
*x*_2_	0.06	0.74	*x*_9_	0	0.50
*x*_3_	0.06	0.74	*x*_10_	0.02	0.71
*x*_4_	0.25	0.10	*x*_11_	0	0.50
*x*_5_	0.09	0.04	*x*_12_	0.06	0.58
*x*_6_	0.17	0.07	*x*_13_	0.04	0.07
*x*_7_	0.41	0.17			

The components with high fuzzy importance values are regarded as weak links in the CGDS system. Enhancing the system's reliability can be achieved by implementing reliability optimization and redundancy design for these components. Moreover, it is crucial to prioritize fault detection and maintenance for these components during equipment integrity management.

## 5 Discussion

In this paper, we propose a quantitative evaluation method for assessing the reliability of urban gas distribution stations. The method employs T-S FTs instead of classical FTs, taking into account the polymorphism of component faults and incorporating linguistic information, such as operational experience or expert knowledge, into logic gates. This approach effectively addresses the limitations of classical approaches [13]. Moreover, the recommended method tackles the challenges of estimating the current fault state value of the basic event under uncertainty conditions and the uncertainty caused by sparse fault sample data by introducing fuzzy group decision-making and Bayesian updating estimation [39]. These enhancements further expand the functionality and applicability of the T-S FFT.

In the present work, we compare the results of reliability analysis for a CGDS based on the extended T-S FFT and the classical FT, as presented in Table 18. It is evident that the classical FT offers limited information, as it only considers two fault states (0,1) and explicit relationships between events. Specifically, the classical FT fails to provide the fuzzy possibilities and occurrence probabilities for the various fault states of the top event. Furthermore, the probability of the top event's fault state being equal to 1, as well as the importance ranking of BEs determined by the classical FT, differ from the results obtained through the extended T-S FFT (refer to Table 19, columns 3 and 5).

**Table 18:** Comparison of system reliability analysis results between extended T-S FFT and Classical FTA.

Reliability data for each fault state of the top event	Extended T-S FFT			Classical FTA	
	*T* = 1	*T* = 0.5	*T* = 0	*T* = 1	*T* = 0
Occurrence probabilities	(7.91, 31.51, 63.63) × 10^−5^	(1.90, 7.29, 15.31) × 10^−5^	(9.9999, 9.9961, 9.9921) × 10^−1^	2.708 × 10^−4^	9.997 × 10^−1^
Fuzzy possibilities	0.755	0.096	0.096	1	0

**Table 19:** Comparison of BE importance measures between extended T-S FFT and Classical FT.

Component	Extended T-S FFT		Classical FTA	
	$I_{F_{u}}^{\left(1\right)}\left(x_{i}\right)$	Ranking	$I_{F_{u}}^{\left(1\right)}\left(x_{i}\right)$	Ranking
*x*_1_	7.80E-01	1	9.99817E-01	1
*x*_2_	7.40E-01	3	9.99810E-01	2
*x*_3_	7.40E-01	3	9.99783E-01	10
*x*_4_	1.00E-01	10	9.99809E-01	3
*x*_5_	4.00E-02	13	9.99776E-01	13
*x*_6_	7.00E-02	11	9.99784E-01	8
*x*_7_	1.70E-01	9	9.99795E-01	5
*x*_8_	7.70E-01	2	9.99793E-01	6
*x*_9_	5.00E-01	7	9.99798E-01	4
*x*_10_	7.10E-01	5	9.99780E-01	12
*x*_11_	5.00E-01	7	9.99781E-01	11
*x*_12_	5.80E-01	6	9.99789E-01	7
*x*_13_	7.00E-02	11	9.99784E-01	9

It is unavoidable that the recommended methods still need further improvement in terms of constructing T-S fuzzy gate description rules and fault diagnostic capabilities. The description rules of T-S fuzzy gate depict the causal relationship between input events and output events. Currently, the construction of the rules primarily relies on expert knowledge and historical data [17,30]. However, in many cases, the available historical data is very limited, or the correlation between the obtained historical data is weak or even irrelevant. Even for relatively small T-S fault trees, the number of rules is high (as shown in Table 4, Table 5, Table 6, Table 7, Table 8), thus the current methods for rule construction present certain challenges.

Based on common sense, when the degree of fault in output events deviates more from that of input events, the assigned fuzzy possibility for the same degree of fault in the output events should be lower in the rules. Moving forward, we will strive to adhere to this fundamental principle and incorporate expert knowledge to develop more rational and objective methods for constructing T-S gate description rules. On the other hand, T-S FFT lack the capability for backward reasoning, whereas Bayesian networks excel in evidence-based reverse probability updates [2]. Exploring how to combine these two approaches to further enhance the system fault diagnosis capability of T-S FFT is also a question that we will investigate in future.

## 6 Conclusions

City gas stations (CGSs) play a crucial role in supplying gas to urban areas, and any shutdowns caused by malfunctions can significantly disrupt the normal work and life of users. Therefore, ensuring the safety and reliability of CGSs is of utmost importance. This paper proposes a systematic and comprehensive methodology for evaluating the reliability of CGSs, taking into account the uncertainties and complexities associated with these stations.

The presented approach constructs T-S fuzzy gates to describe the logical relationships between system events, addressing the polymorphism of faults and the uncertainty of event relationships. Fuzzy numbers are used to depict the severity of component faults, and the impacts of component fault states on the system are considered, resulting in more practical engineering results. Additionally, an improved fuzzy group decision-making approach is applied to estimate the current fault state value of each component of a gas station, and Bayesian estimation models are introduced to calculate the fuzzy probabilities of various fault states of the components.

The effectiveness of the proposed methodology is demonstrated through a case study of a city gas distribution station, highlighting its capacity to analyze data uncertainty. By calculating the failure probabilities of the system and performing T-S fuzzy importance analysis on each component, the vulnerabilities within the system are identified, providing valuable insights for the maintenance management of station equipment. Future work will focus on expanding the method of constructing T-S fuzzy gate descriptive rules and implementing fault diagnosis based on backward reasoning.

## Ethics statement

No ethical approval is needed for this article.

## Data availability statement

All data included in this study are available upon request by contact with the corresponding author.

## CRediT authorship contribution statement

**Daqing Wang:** Writing – review & editing, Writing – original draft, Methodology, Investigation, Funding acquisition, Conceptualization. **Ping Liang:** Methodology, Formal analysis. **Tingting Luo:** Writing – original draft, Methodology. **Haihong Yu:** Validation.

## Declaration of competing interest

The authors declare the following financial interests/personal relationships which may be considered as potential competing interests: Daqing Wang reports financial support was provided by 10.13039/501100002865Chongqing Science and Technology Bureau. If there are other authors, they declare that they have no known competing financial interests or personal relationships that could have appeared to influence the work reported in this paper.
